# SamSelect: a sample sequence selection algorithm for quorum planted motif search on large DNA datasets

**DOI:** 10.1186/s12859-018-2242-y

**Published:** 2018-06-18

**Authors:** Qiang Yu, Dingbang Wei, Hongwei Huo

**Affiliations:** School of Computer Science and Technology, Xidian University, Xi’an, 710071 China

**Keywords:** Quorum planted motif search, Sample sequences, Transcription factor binding sites

## Abstract

**Background:**

Given a set of *t n*-length DNA sequences, *q* satisfying 0 < *q* ≤ 1, and *l* and *d* satisfying 0 ≤ *d* < *l* < *n*, the quorum planted motif search (qPMS) finds *l*-length strings that occur in at least *qt* input sequences with up to *d* mismatches and is mainly used to locate transcription factor binding sites in DNA sequences. Existing qPMS algorithms have been able to efficiently process small standard datasets (e.g., *t* = 20 and *n* = 600), but they are too time consuming to process large DNA datasets, such as ChIP-seq datasets that contain thousands of sequences or more.

**Results:**

We analyze the effects of *t* and *q* on the time performance of qPMS algorithms and find that a large *t* or a small *q* causes a longer computation time. Based on this information, we improve the time performance of existing qPMS algorithms by selecting a sample sequence set *D*’ with a small *t* and a large *q* from the large input dataset *D* and then executing qPMS algorithms on *D*’. A sample sequence selection algorithm named SamSelect is proposed. The experimental results on both simulated and real data show (1) that SamSelect can select *D*’ efficiently and (2) that the qPMS algorithms executed on *D*’ can find implanted or real motifs in a significantly shorter time than when executed on *D*.

**Conclusions:**

We improve the ability of existing qPMS algorithms to process large DNA datasets from the perspective of selecting high-quality sample sequence sets so that the qPMS algorithms can find motifs in a short time in the selected sample sequence set *D*’, rather than take an unfeasibly long time to search the original sequence set *D*. Our motif discovery method is an approximate algorithm.

## Background

DNA motif discovery is a key factor in locating regulatory elements (e.g., transcription factor binding sites) in DNA sequences [1–4]. The quorum planted motif search (qPMS) [5, 6], a widely studied formulation for motif discovery, defines a motif as an *l*-length string (*l*-mer) *m* that occurs in at least *qt* out of *t n*-length (*n* > *l*) input sequences with up to *d* (0 ≤ *d* < *l*) mismatches, where *q* (0 < *q* ≤ 1) is the proportion of the input sequences containing motif occurrences; *m* and its occurrences in the sequences are called an (*l*, *d*) motif and its instances, respectively. Given a set of *t n*-length DNA sequences *D =* {*s*_1_, *s*_2_, …, *s*_*t*_} containing a motif *m* and the parameters *l*, *d* and *q* describing *m*, the task of qPMS is to find all (*l*, *d*) motifs present in *D* such that *m* must exist in the found motifs.

qPMS is NP-complete [7]. Over the past two decades, there have been many studies on qPMS algorithms [8–11]. The qPMS algorithms are based on searching possible combinations of motif instances or possible candidate motifs and are either sample driven or pattern driven. The sample-driven qPMS algorithms, such as WINNOWER [5], DPCFG [12] and RecMotif [13], have an initial search space of (*n* – *l* + 1)^*t*^*t*-tuples (*x*_1_, *x*_2_, …, *x*_*t*_) in the case of *q* = 1; each *t* tuple is composed of *t l*-mers from *t* input sequences, i.e., a group of possible motif instances. The pattern-driven qPMS algorithms have an initial search space of 4^*l*^ candidate motifs and verify if each candidate motif is an (*l*, *d*) motif. Because of the much smaller initial search space, the pattern-driven qPMS algorithms usually exhibit better time performance than the sample-driven qPMS algorithms.

The time performance of the pattern-driven qPMS algorithms depends mainly on two aspects: the number of candidate motifs and the efficiency of candidate motif verification. To speed up candidate motif verification, the suffix tree-based pattern driven (stpd) qPMS algorithms, such as Speller [14], Weeder [15], RISOTTO [16] and FMotif [17], construct a suffix tree of input sequences. The basic procedure for verifying a candidate motif *m* is then as follows: match *m* along different paths from the suffix tree root and record the current number of mismatches *e* on each path; if *e* is greater than *d*, then terminate the match on the corresponding path; and if the *l*-length paths with *e* ≤ *d* correspond to a group of strings that can span at least *qt* input sequences, then *m* is determined to be an (*l*, *d*) motif.

With a focus on reducing the number of candidate motifs, some algorithms combine the sample-driven and pattern-driven approaches. These are called sample-pattern-driven (spd) qPMS algorithms. In the sample-driven phase, these algorithms use *t* – *qt* + *h* reference sequences, which must contain at least *h* motif instances, and traverse all the *h*-tuples (*x*_1_, *x*_2_, …, *x*_*h*_) in these reference sequences. An *h*-tuple consists of *h l*-mers from different reference sequences, i.e., a group of *h* possible motif instances. In the pattern-driven phase, these algorithms generate common *d*-neighbors of each *h*-tuple (a *d*-neighbor of an *h*-tuple is an *l*-mer *y* such that the Hamming distance between *y* and each *l*-mer *x*_*i*_ in the *h*-tuple is less than or equal to *d*), and take them as candidate motifs to verify one by one. The existing spd qPMS algorithms can be classified according to the different values of *h*, as follows: PMSP [18] and PMSprune [6] have *h* = 1, PairMotif [19], qPMS7 [20] and TravStrR [21] have *h* = 2, iTriplet [22] and PMS5 [23] have *h* = 3, and PMS8 [24] and qPMS9 [25] have *h* ≥ 3.

The existing qPMS algorithms currently perform well when processing traditional standard DNA datasets [5] (e.g., *t* = 20, *n* = 600), even for challenging (*l*, *d*) problem instances [26]. However, these algorithms encounter bottlenecks when processing large DNA datasets, such as the ChIP-seq datasets [9, 27], which typically contain thousands of DNA sequences or even more. ChIP-seq datasets enable the identification of transcription factor binding sites within the genome but present a significant computational challenge for qPMS. First, the sample-driven qPMS algorithms undergo a combinatorial explosion because the search space grows exponentially with the number *t* of DNA sequences. Second, for the stpd qPMS algorithms, the running time shows quadratic growth as *t* increases and also increases as *q* decreases (see the analysis in the section Why to Select Sample Sequences). Third, for the spd qPMS algorithms, there are too many *h*-tuples to be considered in the *t* – *qt* + *h* reference sequences, greatly extending the time required. Therefore, it is necessary to accelerate the existing qPMS algorithms for large DNA datasets.

As described above, the time performance of the qPMS algorithms is affected by both the number *t* of input sequences and the proportion *q* of the input sequences containing motif instances; specifically, a large *t* or a small *q* will increase the computation time for both the stpd and the spd qPMS algorithms. Consider a dataset *D* of a motif *m* such that there are *qt* sequences containing instances of *m* in a total of *t* sequences and a subset *D*’ of *D* such that there are *q*’*t*’ sequences containing instances of *m* in a total of *t*’ sequences, satisfying 0 < *t*’ < *t* and 1 ≥ *q*’ > *q* > 0. It is not difficult to find that when a qPMS algorithm is executed on *D* and *D*’ separately, the motif *m* can be found in both cases, and the running time on *D*’ can be significantly smaller than that on *D*. Based on this consideration, given a large DNA dataset *D*, one way to effectively improve the time performance of qPMS algorithms is to select a portion of the sequences from *D* to form a sample sequence set *D*’, making the proportion of the sequences containing motif instances higher in *D*’ than in *D*, and then execute qPMS algorithms on *D*’ to perform motif discovery.

In this paper, we analyze why the selection of sample sequences for the qPMS algorithms is important. Then, we propose a method of selecting sample sequences. Additionally, we use both simulated data and real data to validate the ability of the qPMS algorithms to perform motif discovery on the selected sample sequences, i.e., whether they can find the implanted or real motifs in a significantly shorter time.

## Methods

### Why to select sample sequences

The notations frequently used in this paper are summarized in Table 1.

**Table 1:** Notations used in this paper

Notation	Explanation
*|x|*	The length of a string or the size of a set.
Σ	The DNA alphabet, Σ = {A, C, G, T}.
*l-mer*	An *l*-length string over Σ.
*s*[*i*]	The *i*th character in the string *s*.
*s*[*i*..*j*]	A substring of the string *s* from the *i*th position to the *j*th position.
*s*∙*s*’	The concatenation of two strings *s* and *s*’.
*x* ∈_*l*_*s*	The string *x* is an *l*-length substring of the string *s*. In other words, *x* is an *l*-mer in the string *s*.
*x* ∈_*l*_*D*	The string *x* is an *l*-length substring of the sequence set *D*. In other words, there exists *s* ∈ *D* such that *x* ∈_*l*_*s*.
*D =* {*s*_1_, *s*_2_, …, *s*_*t*_}, *t*, *n*, *q*, *l*, *d*	Notations for the input. *D* is the input DNA sequence set, where each sequence *s*_*i*_ is an *n*-length string over Σ; *t* = |*D*|; *n* = |*s*_*i*_| for 1 ≤ *i* ≤ *t*; *q* is the proportion of the input sequences containing motif instances in *D*; *l* is the motif length and *d* is the maximum number of mismatches between a motif and its instance.
*D*’, *t*’, *q*’	Notations for the output. *D*’ is a sample sequence set selected from *D*, i.e., *D*’ ⊂ *D*; *t*’ = |*D*’|; *q*’ is the proportion of the input sequences containing motif instances in *D*’.
*count*_*k*_(*x*)	The count (number of occurrences) of a string *x* in *D* with up to *k* mismatches, represented by (4).
*count*(*x*)	The count (number of occurrences) of a string *x* in *D*.
*d*_*H*_(*y*, *x*)	The Hamming distance between two strings *y* and *x* of equal length.
*B*_*k*_(*x*)	The set of *k*-neighbors of a string *x*, i.e., the set of strings with Hamming distance no more than *k* from *x*. *B*_*k*_(*x*) = {*y*: *y* ∈ Σ^|*x*|^, *d*_*H*_(*y*, *x*) ≤ *k*}.
*stn*(*y*)	The integer obtained by conversion from a string *y* over Σ. The characters A, C, G and T are converted to binary numbers 00, 01, 10 and 11, respectively. Because of the need to compute *count*_*k*_(*y*), *y* is first reversed and then converted to an integer. For example, if *y* = AC, then *y* is converted to the binary number 0100, i.e., the decimal number 4.

Fixing (*l*, *d*) and the length *n* of a single sequence, we analyze the effects of the number *t* of input sequences and the proportion *q* of the input sequences containing motif instances on the time performance of qPMS algorithms. We analyze the stpd and the spd qPMS algorithms.

The stpd qPMS algorithms construct a suffix tree of *t n*-length input sequences [14]. In the tree, each edge is labeled with a non-empty substring of the input sequences, and each node *v* corresponds to a string *str*_*v*_ representing the concatenation of the substrings on the path from the root of tree to *v*. If *v* is a leaf, then *str*_*v*_ is a suffix of input sequences; otherwise, *str*_*v*_ is a common prefix of the suffixes represented by all leaves under *v*. The suffix tree has exactly *tn* leaves, representing *tn* suffixes of input sequences. For each node *v* of the tree, the IDs of sequences in which *str*_*v*_ occurs exactly are stored by using a vector of *t* bits for good storage efficiency.

In addition to the suffix tree, these algorithms also use a pattern tree, a complete quadtree of depth *l* representing all the patterns over Σ with length ranging from 1 to *l*. Then, they perform a depth-first search on the pattern tree. When visiting a node *v* corresponding to a pattern *p*, they use the suffix tree to obtain the IDs of sequences in which all *d*-neighbors of *p* occur exactly, i.e., the IDs of sequences in which *p* occurs with up to *d* mismatches. If the number of the sequence IDs obtained is greater than or equal to *qt* and the length of *p* is less than *l*, they continue to visit the children of *v* corresponding to the patterns *pb* (*b* ∈ Σ) and otherwise prune the subtree of *v*. Finally, they output all the *l*-length patterns that span at least *qt* sequences.

The time and space complexity of the stpd qPMS algorithms can be evaluated as follows [14]. The suffix tree of *t n*-length sequences has *tn* leaves and thus up to *tn* nodes of *l*-length strings; for each such node *v* in the suffix tree, at most |*B*_*d*_(*str*_*v*_)| patterns in the pattern tree have up to *d* mismatches with *str*_*v*_; for each such pattern *y*, when it is verified as a candidate motif, the node *v* needs to be visited once, and the binary OR operation is executed on the vector of *t* bits in *O*(*t*) time. Therefore, the time complexity is *O*(*t*^2^*n*|*B*_*d*_(*str*_*v*_)|), which is approximately *O*(*t*^2^*nl*^*d*^4^*d*^). Since a vector of *t* bits is stored in each of *O*(*tn*) nodes of the suffix tree, the space complexity is *O*(*t*^2^*n*/*w*), where *w* is the word size of the computer.

We find that *t* has a strong effect on both the time and space performance of the stpd qPMS algorithms, i.e., both the running time and the storage space show quadratic growth as *t* increases. Furthermore, although *q* does not appear in the time complexity evaluated above, it also affects the time performance because it affects the pruning efficiency when searching the pattern tree. As described above, the subtree of a node *v* corresponding to a pattern *p* that cannot span at least *qt* sequences is pruned. If *q* is small, then *p* has a higher probability *P*_span_ of spanning at least *qt* sequences (*P*_span_ is calculated by (1), where *P*_*d*_ is the probability that the Hamming distance between two random *l*-mers is less than or equal to *d*), which is detrimental to pruning. Therefore, the smaller the value of *q*, the higher is the computational time of the stpd qPMS algorithms.

1 $$ {P}_{\mathrm{span}}=\sum \limits_{i= qt}^t\left(\begin{array}{c}t\\ {}i\end{array}\right){\left(1-{\left(1-{P}_d\right)}^{\left(n-l+1\right)}\right)}^i{\left({\left(1-{P}_d\right)}^{\left(n-l+1\right)}\right)}^{t-i} $$

2 $$ {P}_d=\sum \limits_{i=0}^d\left(\begin{array}{c}l\\ {}i\end{array}\right)\frac{{\left(\left|\sum \right|-1\right)}^i}{{\left|\sum \right|}^l} $$

The time performance of the spd qPMS algorithms depends mainly on the number of generated candidate motifs. These algorithms use all *h*-tuples in *t* – *qt* + *h* reference sequences to generate candidate motifs. That is, they must consider all possible combinations of *h* reference sequences in *t* – *qt* + *h* reference sequences; the number of possible combinations is denoted by *N*_*com*_ and calculated by (3). For a given algorithm, the value of *h* (*h* ≥ 1) is generally fixed, so *N*_*com*_ is mainly affected by *t* and *q*. Obviously, when *t* increases or *q* decreases, *N*_*com*_ will increase, leading to more candidate motifs and a higher computation time.

3 $$ {N}_{com}=\left(\begin{array}{c}t- qt+h\\ {}h\end{array}\right)=\frac{\prod \limits_{i=1}^h\left(t- qt+i\right)}{h!} $$

Based on the above analysis, both *t* and *q* have the same effect on the stpd qPMS algorithms as on the spd qPMS algorithms: a large *t* or a small *q* will increase the computation time. Large DNA datasets, such as ChIP-seq datasets (see Tables 2 and 3), typically contain thousands DNA sequences or even more; that is, *t* is very large. On the other hand, the proportion of sequences containing motif instances is not large, that is, *q* is small. The two aspects make qPMS algorithms too time consuming to process large DNA datasets.

**Table 2:** Real datasets selected from the ENCODE TF ChIP-seq data

Dataset	Motif	(*l*, *d*)	*t*	*q*
egr1	CCGCCCCCGCA	(11, 3)	15,400	0.68
elf1	AACCCGGAAGT	(11, 3)	8611	0.54
hnf4	GGGTCAAAGTCCA	(13, 4)	11,045	0.53
myc	ACCACGTGCTC	(11, 3)	4542	0.49
nfy	ACTAACCAATCAG	(13, 4)	9781	0.44
sp1	GGGGCGGGG	(9, 2)	14,779	0.52
srf	TGACCATATATGGTC	(15, 5)	4903	0.36
yy1	CGGCCATCT	(9, 2)	2077	0.49

**Table 3:** Real datasets in the mESC data

Dataset	Motif	(*l*, *d*)	*t*	*q*
c-Myc	GCACGTGGC	(9, 2)	3422	0.60
CTCF	CCACCAGGGGGCG	(13, 4)	39,601	0.58
Esrrb	GGTCAAGGTCA	(11, 3)	21,644	0.54
Klf4	GGGTGTGGC	(9, 2)	10,872	0.61
Nanog	CCTTGTCATGC	(11, 3)	10,342	0.26
n-Myc	GCACGTGGC	(9, 2)	7181	0.57
Oct4	CATTGTTATGCAAAT	(15, 5)	3775	0.29
Smad1	CCTTTGTTATGCA	(13, 4)	1126	0.36
Sox2	CATTGTTATGCAAAT	(15, 5)	4525	0.39
STAT3	TTCCCGGAA	(9, 2)	2546	0.61
Tcfcp2I1	CCGGTTCAAACCG	(13, 4)	26,907	0.29
Zfx	GCTAGGCCGCG	(11, 3)	10,336	0.49

One way to effectively improve the time performance of qPMS algorithms is to select a sample sequence set *D*’ with a larger proportion of sequences containing motif instances from the given dataset *D* and then to execute qPMS algorithms on *D*’ to perform motif discovery. Accordingly, the problem to be solved is described as follows.

#### Sample sequence selection problem

Given a set of *t n*-length DNA sequences *D =* {*s*_1_, *s*_2_, …, *s*_*t*_} containing instances of a motif *m*, along with the parameters *l*, *d* and *q* describing *m* (see Table 1 for the explanation of these parameters), the task is to select a portion of the sequences from *D* to form a sample sequence set *D*’ (let *t*’ = |*D*’|, and let *q*’ be the proportion of sequences containing instances of *m* in *D*’), so that *t*’ < *t* and *q*’ > *q*.

### How to select sample sequences

#### Basic concept

Because of the conservation of DNA motifs, the instances of a particular motif are similar to each other. Thus, if a substring *x* in the input sequences overlaps a motif instance, the occurrence frequency of *x* is generally higher than that of a substring *y* with |*y*| = |*x*| in the background sequences. Based on this difference in frequency, our basic idea is to convert the problem of selecting sample sequences containing motif instances into the problem of selecting sample sequences containing high-frequency substrings. That is, we test whether a sequence contains a high-frequency substring to determine whether the sequence contains a motif instance.

Since most of the motif instances are similar but not exactly the same, the occurrence frequency of a substring *x* is evaluated by the count of *x* in *D* with up to *k* mismatches, denoted by *count*_*k*_(*x*), i.e., the number of substrings *y* in *D* satisfying *d*_*H*_(*y*, *x*) ≤ *k*. Notably, the time complexity of computing *count*_*k*_(*x*) for a substring *x* grows dramatically as *k* increases; moreover, we need to compute *count*_*k*_(*x*) for all substrings of a specified length *w* in the input sequences. Therefore, the value of *k* cannot be large if good time complexity is to be achieved. When *k* is small, the length *w* should also be small to obtain enough substrings overlapping motif instances.

The length *w* is generally smaller than the motif length *l*, and a motif instance in a sequence may produce multiple overlapped high-frequency *w*-mers. Therefore, after fetching high-frequency *w*-mers, a step is needed to combine multiple overlapped *w*-mers into one high-frequency substring. The length of the combined high-frequency substrings may not be equal but is generally greater than *l*. A high-frequency substring is expected to cover a motif instance.

Furthermore, the obtained high-frequency substrings need to be grouped. To guarantee a large value of *q*’, a sample sequence set is expected to contain only instances of a single motif. However, the input sequences may contain multiple motifs and the disturbance of random high-frequency substrings; that is, in general, the obtained high-frequency substrings are composed of instances of multiple motifs and some random high-frequency substrings. Therefore, we use a clustering method to divide the obtained high-frequency substrings into groups and thus may obtain two or more high-quality sample sequence sets so that a sample sequence set exists corresponding to the motif to be found.

Based on these considerations, SamSelect consists of the following three steps: i) word count with mismatches, used to fetch high-frequency *w*-mers; ii) high-frequency substring obtainment, used to obtain high-frequency substrings by combining overlapped *w*-mers; and iii) high-frequency substring grouping, used to obtain sample sequence sets by clustering high-frequency substrings.

#### Word count with mismatches

We compute *count*_*k*_(*x*) for all *w*-mers *x* in the input sequences. Given a *w*-mer *x*, *count*_*k*_(*x*) is represented as

4 $$ {count}_k(x)=\sum \limits_{y{\in}_wD}{I}_y, $$

where *I*_*y*_ is an indicator variable and it is 1 if *d*_*H*_(*y*, *x*) ≤ *k*, 0 otherwise.

Our method for computing *count*_*k*_(*x*) is based on the count operation (computing the number of occurrences of a string *y* in *D*, i.e., *count*(*y*)) of FM-Index [28]. That is, *count*_*k*_(*x*) is converted into the sum of the number of occurrences of all *k*-neighbors of *x*:

5 $$ {count}_k(x)=\sum \limits_{y\in {B}_k(x)} count(y). $$

FM-Index is a self-indexed data structure. Let [*L*_*y*_, *R*_*y*_] denote the ranking interval of the suffixes of input sequences prefixed by a string *y*. With [*L*_*y*_, *R*_*y*_], *count*(*y*) = *R*_*y*_*– L*_*y*_ *+* 1 can be obtained immediately. The process of computing [*L*_*y*_, *R*_*y*_] is to traverse *w* characters of *y* from right to left (i.e., backward search); when the *i*th (1 ≤ *i* ≤ *w*) character *y*[*i*] is visited, the interval [*L*_*φ*_, *R*_*φ*_] for *φ = y*[*i*..*w*] is obtained in *O*(log|Σ|) time based on the interval [*L*_*φ*’_, *R*_*φ*’_] for *φ*’ *= y*[*i* + 1..*w*] through FM-Index. Thus, *count*(*y*) is computed in *O*(*w*log|Σ|) time.

The count of a single *w*-mer can be computed efficiently with FM-Index, but if we obtain *count*_*k*_(*x*) by independently computing the count of each *w*-mer in *B*_*k*_(*x*), then the backward search on the common suffixes of *w*-mers in *B*_*k*_(*x*) will be performed repeatedly. For example, when computing *count*_1_(*x*) for a 3-mer *x* = ACG, if we independently compute the counts of the four 3-mers ACG, CCG, GCG and TCG in *B*_1_(*x*), then the backward search on the common suffix CG will be performed four times. Moreover, our goal is to obtain *count*_*k*_(*x*) for all *w*-mers *x* in the input sequences, making the number of repeated backward searches even larger.

**Fig. 1:**
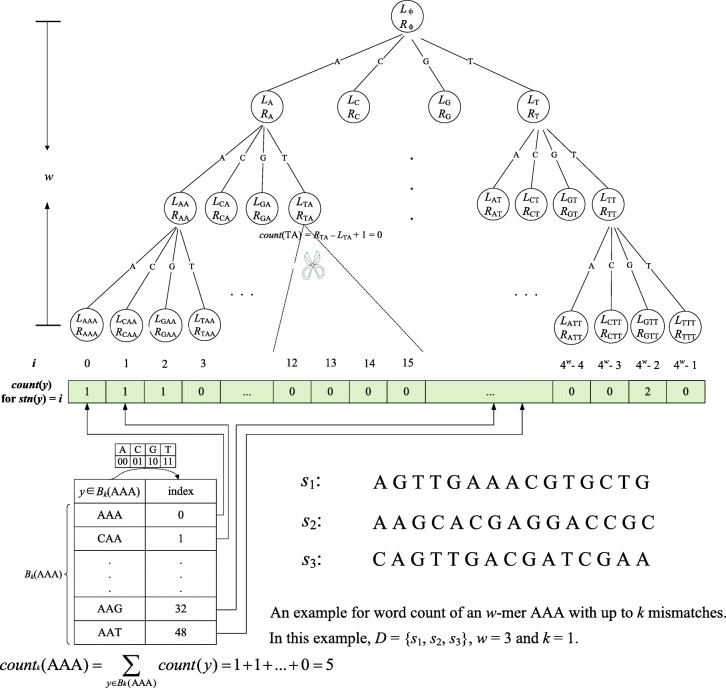
Illustration of word count with mismatches. This figure shows an illustration of word count with up to *k* mismatches

To address this problem, we design a method to minimize the number of repeated backward searches. As shown in Fig. 1, we first efficiently compute the values of *count*(*y*) for all *w*-mers *y* in the input sequences by using Algorithm 1 and store them in a Table *T* of size 4^*w*^, where *T*[*i*] stores the value of *count*(*y*) for the *w*-mer *y* with *stn*(*y*) = *i*; then, we obtain *count*_*k*_(*x*) for a given *w*-mer *x* by querying *T* |*B*_*k*_(*x*)| times and summing *T*[*stn*(*y*)] for each *y* in *B*_*k*_(*x*). In Algorithm 1, we obtain *T* by searching a quadtree of depth *w*. The leaves and internal nodes of the quadtree correspond to all *w*-length strings over Σ and their common suffixes, respectively. All elements in *T* are initialized to zero; in searching the quadtree, when the value of *count*(*y*) for a *w*-mer *y* is greater than zero, *T*[*stn*(*y*)] is updated to *count*(*y*).

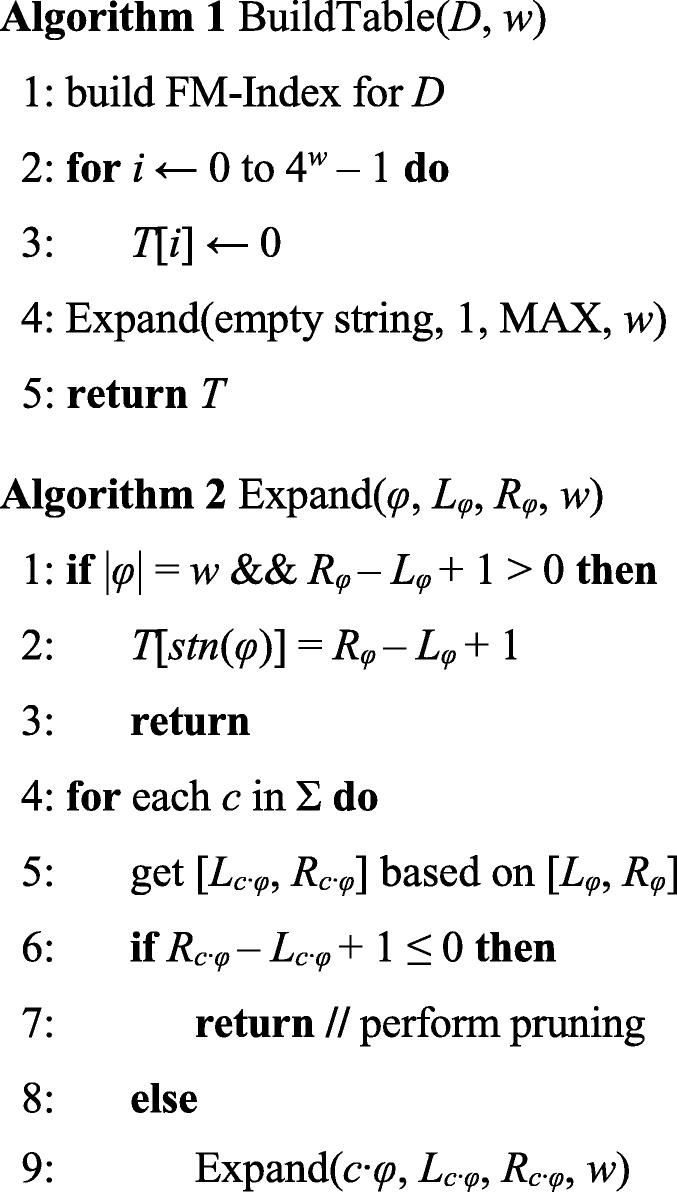



Algorithm 1 is able to minimize the number of repeated backward searches. When an arbitrary node *v* of the quadtree is being visited (let *φ* be the string corresponding to *v*), the interval [*L*_*φ*’_, *R*_*φ*’_] for *φ*’ = *φ*[2..*|φ|*] has already been obtained, and only *O*(log|Σ|) time is needed to obtain the interval [*L*_*φ*_, *R*_*φ*_] for *φ*. Therefore, for all strings with a common suffix *φ*, the backward search on the suffix *φ* is only executed once. Moreover, we use pruning technology in the search process. Once *count*(*φ*) for a string *φ* that corresponds to a node *v* is 0, the subtree of *v* is pruned.

To guarantee good space and time performance of word count with up to *k* mismatches, it is necessary to select appropriate values of *w* and *k*. Except for building FM-Index, which is not affected by *w* and *k*, the space complexity is *O*(4^*w*^), which is mainly used to store the Table *T*. The time complexity *T*_count_ depends on two parts, *T*_1_ and *T*_2_. *T*_1_ is involved in building *T* by visiting every node of the *w*-depth quadtree in the worst case. *T*_2_ is used to compute *count*_*k*_(*x*) for each *w*-mer *x* in *t n*-length sequences by querying *T* |*B*_*k*_(*w*-mer)| times.

6 $$ {\displaystyle \begin{array}{l}{T}_{\mathrm{count}}=O\left({T}_1+{T}_2\right)\\ {}\kern2em =O\left(\sum \limits_{i=0}^w{4}^w\log \left|\Sigma \right|+ tn\left|{B}_k\left(w-\mathrm{mer}\right)\right|\right)\\ {}\kern2em =O\left(\sum \limits_{i=0}^w{4}^w\log \left|\Sigma \right|+ tn\sum \limits_{i=0}^k\left(\begin{array}{c}w\\ {}i\end{array}\right){\left(\left|\Sigma \right|-1\right)}^i\right)\end{array}} $$

Because *k* affects the time *T*_2_, it is expected to be kept as small as possible; on the other hand, since the instances of a particular motif are a group of substrings similar to each other, it is more meaningful that *k* is greater than or equal to 1. The value of *w* affects both the space and time performance of the word count with up to *k* mismatches. According to empirical studies, *w* should be less than 15 to guarantee good performance by a personal computer. In SamSelect, we set *w* and *k* to 12 and 1, respectively. With this setting, in addition to the guarantee of good space and time performance, we would also like to obtain more motif information, as the probability analysis shows that *count*_1_(12-mer) for a motif instance is significantly larger than that for a background substring [29].

#### High-frequency substring obtainment

We use high-frequency substrings in input sequences to represent the corresponding sequences, and make the following considerations for obtaining high-frequency substrings. First, we select the *w*-mers *x* in input sequences with *count*_*k*_(*x*) greater than a certain threshold *f*, combine the overlapped *w*-mers to one substring and store the substrings of length greater than or equal to *l* in a set *A*. Second, to guarantee good time performance of the substring clustering in the next step, we set the total number of substrings to no more than 5000, which is much larger than the number of outputted sample sequences; if we obtain more than 5000 substrings, we will increase *f* repeatedly by a small amount. Third, we need to segment long high-frequency substrings because they may contain instances of two or more adjacent different motifs. This division guarantees that the substrings in a particular group correspond to the instances of the same motif; after segmentation, we store the substrings of length greater than or equal to *l* to a set *A*’.

**Fig. 2:**
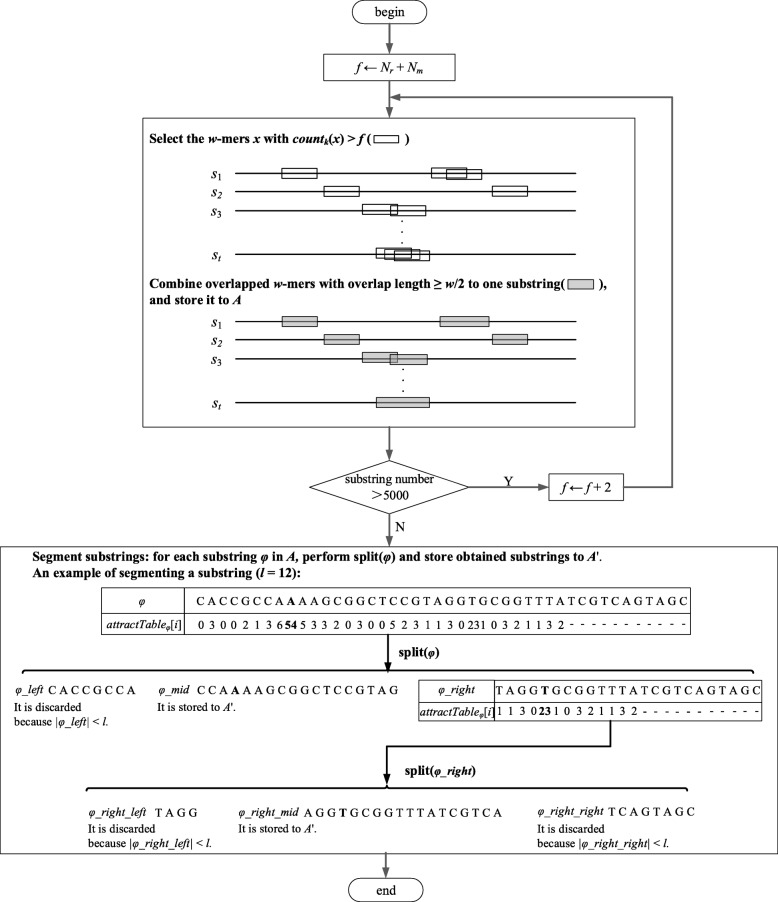
Illustration of obtaining high-frequency substrings. This figure illustrates the process of obtaining high-frequency substrings. *N*_*r*_ and *N*_*m*_ are *count*_*k*_(*w*-mer) for a background substring and a motif instance in the random case, respectively

The overall process of this step is shown in Fig. 2. The initial value of threshold *f* is set to the sum of *N*_*r*_ and *N*_*m*_, where *N*_*r*_ and *N*_*m*_ are *count*_*k*_(*w*-mer) for a background substring and a motif instance for a random case, respectively; the calculation method of *N*_*r*_ and *N*_*m*_ is given in [29]. For any two overlapped *w*-mers, if the length of the overlap is greater than or equal to *w*/2, we combine the two *w*-mers into one substring. Notably, some substrings are obtained by combining more than two overlapped *w*-mers (e.g., the substring of *s*_*t*_ in Fig. 2).

Next, we describe how to segment substrings. We first give some definitions. A |*φ*| – *l* + 1 size table denoted by *attractTable*_*φ*_ is built for each substring *φ* in *A*. To explain this table, we define the distance *dis*(*φ*, *φ*’) between two given substrings *φ* and *φ*’ as the minimum Hamming distance between two *l*-mers *x* ∈_*l*_*φ* and *x*’ ∈_*l*_*φ*’; *dis*(*φ*, *φ*’) is calculated by (7). The *i*th element of the table *attractTable*_*φ*_[*i*] is calculated by (8), where *minPos*_*φ*_(*φ*’) is the set of all positions of the *l*-mers in *φ* leading to *dis*(*φ*, *φ*’).

7 $$ dis\left(\varphi, {\varphi}^{\hbox{'}}\right)=\underset{x{\in}_l\varphi, x\hbox{'}{\in}_l\varphi \hbox{'}}{\min }{d}_H\left(x,{x}^{\hbox{'}}\right) $$

8 $$ {attractTable}_{\varphi}\left[i\right]=\left|\left\{{\varphi}^{\hbox{'}}:{\varphi}^{\hbox{'}}\in A-\left\{\varphi \right\},i\in {minPos}_{\varphi}\left({\varphi}^{\hbox{'}}\right)\right\}\right| $$

9 $$ {minPos}_{\varphi}\left({\varphi}^{\hbox{'}}\right)=\underset{1\le i\le \left|\varphi \right|-l+1}{\arg \min } dis\left(\varphi \left[i\dots i+l-1\right],{\varphi}^{\hbox{'}}\right) $$

The process of segmenting a substring *φ* is given in Algorithm 3. Let *x* be the *l*-mer in *φ* with the position of the maximum element in *attractTable*_*φ*_. Since some deviations may occur between the position of *x* and that of the corresponding motif instance, we cut out *x* from *φ* and form a new substring by extending up to 3 characters from both the left and the right side of *x*. After cutting out *x*, if the length of the remaining left/right part of *φ* is still greater than or equal to *l*, we recursively segment the remaining left/right part of *φ*.

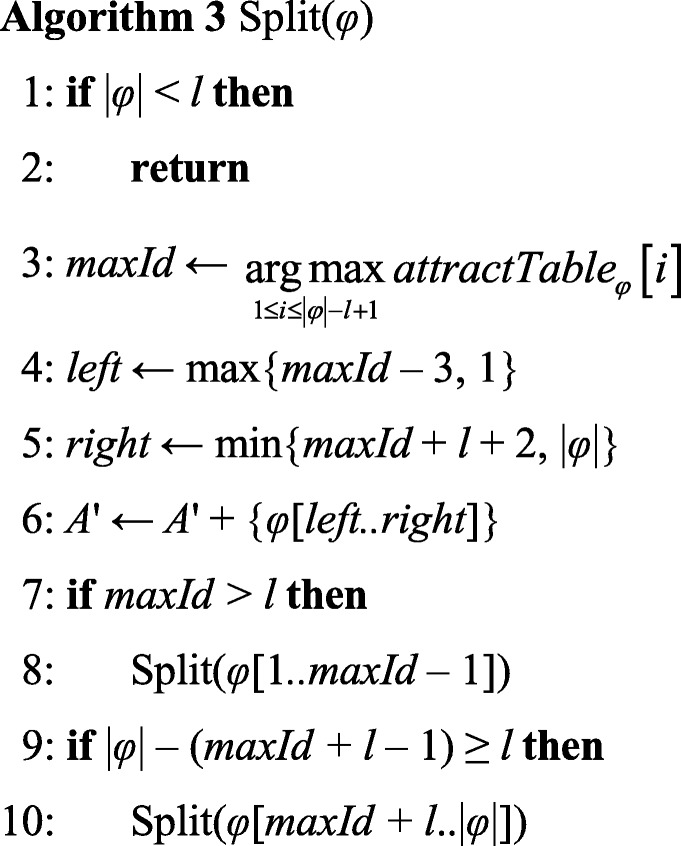



The computation time of this step is mainly determined by the following two aspects. First, we scan all *w*-mers in the entire dataset in *O*(*tn*) time to obtain the initial high-frequency substrings and store them to the set *A*. Second, in segmenting substrings, we need to calculate the distance between each pair of substrings in *A* in *O*(*L*^2^) time, where *L* is the average length of the substrings in *A*. Therefore, the time complexity of this step is *O*(*tn* + |*A*|^2^ *L*^2^).

#### High-frequency substring grouping

We mainly use the clustering method to obtain sample sequence sets. The process is described in Algorithm 4, which includes three stages.

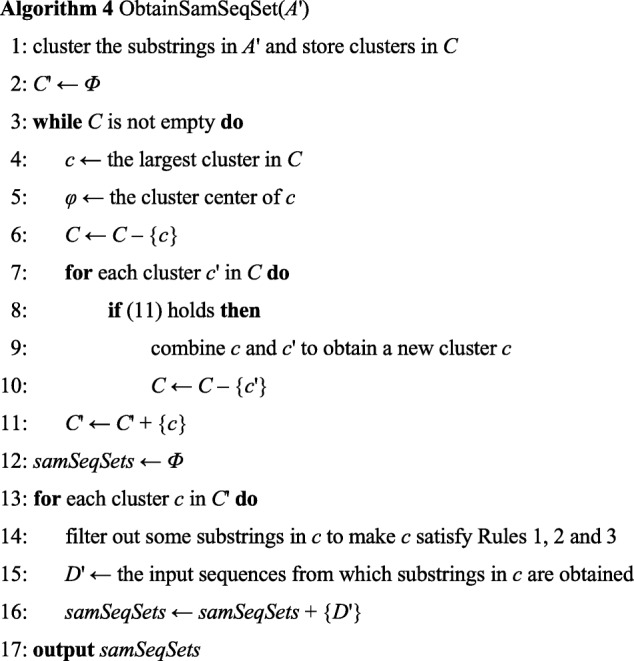



In the first stage (line 1), we cluster the high-frequency substrings to distinguish substrings corresponding to different motifs. The AP algorithm [30] is used for clustering; it can automatically determine the number of clusters and obtain cluster centers. For each cluster, we take the cluster center as the substring that is most similar to the motif and use it to filter out random high-frequency substrings in the cluster. In clustering, the similarity *sim*(*φ*, *φ*’) between two substrings *φ* and *φ*’ is evaluated as follows.

10 $$ sim\left(\varphi, {\varphi}^{\hbox{'}}\right)=\left\{\begin{array}{cc}- dis\left(\varphi, {\varphi}^{\hbox{'}}\right),& \mathrm{if}\  dis\left(\varphi, {\varphi}^{\hbox{'}}\right)\le 2d\\ {}- dis\left(\varphi, {\varphi}^{\hbox{'}}\right)\times 10,& \mathrm{otherwise}\end{array}\right. $$

In the second stage (lines 2 to 11), the resulting clusters are combined, since multiple clusters may correspond to the same motif. For two clusters *c* and *c*’ (|*c*| ≥ |*c*’|), we use the cluster center *φ* of *c* to compare each substring *φ*’ in *c*’; in terms of (11), if the number of *φ*’ satisfying *dis*(*φ*, *φ*’) ≤ *d* is significantly larger than the number under random case *P*_*d*_|*c*’|, we combine *c* and *c*’. Multiple clusters are combined by using a greedy strategy.

11 $$ \left|\left\{{\varphi}^{\hbox{'}}:{\varphi}^{\hbox{'}}\in {c}^{\hbox{'}}, dis\left(\varphi, {\varphi}^{\hbox{'}}\right)\le d\right\}\right|>{P}_d\left|{c}^{\hbox{'}}\right|+20\%\left|{c}^{\hbox{'}}\right| $$

In the third stage (lines 12 to 17), we obtain sample sequence sets. For each cluster *c*, we sort the substrings in *c* in ascending order according to their distance from the cluster center and update *c* by keeping the first *t*’ substrings. The value of *t*’ is specified by the user and should be less than or equal to the maximum number of sequences containing motif instances *qt*. Then, to maximize the possibility that *c* corresponds to a set of motif instances, we use the following three rules in turn to test *c* and filter out a portion of substrings to make *c* satisfy these rules. Thus, the final value of *t*’ may be less than the specified value. Finally, for each cluster *c*, after filtering, we obtain a sample sequence set *D*’ consisting of the input sequences from which substrings in *c* are obtained. If we obtain two or more sample sequence sets, we rank them in descending order by size, since a large sample sequence set is more likely to contain a highly conserved motif.

### Rule 1

The distance between any two substrings in *c* is less than or equal to 2*d*.

### Rule 2

The distance between each substring in *c* and the cluster center is less than or equal to 3*d*/2.

The reason for adopting these two rules is as follows. For any two motif instances, their Hamming distance is less than or equal to 2*d*. The cluster center usually contains a motif instance of high conservation that is close to the motif and at distance < *d* from the motif. Therefore, a more stringent distance constraint (≤ 3*d*/2) should be observed between each substring in *c* and the cluster center.

### Rule 3

The set *c* is a motif set.

The set *c* satisfying Rule 1 is called a *pairwise bounded set*. If *c* is a set of motif instances, a consensus *m* should exist such that the distance between *m* and each substring in *c* is less than or equal to *d*; such set *c* is called a *motif set*. A pairwise bounded set that is not a motif set is called a *decoy set*.

The work of Boucher and King [31] shows a clear difference between the weight of motif sets and that of decoy sets (the weight is calculated by (12)), so the majority of motif sets and decoy sets can be distinguished with statistical methods. Specifically, for a given pairwise bounded set *c*, if *w*(*c*) ≤ *a*_*m*_ or *w*(*c*) ≥ *a*_*d*_, where *a*_*m*_ and *a*_*d*_ (*a*_*m*_ < *a*_*d*_) are two thresholds obtained by statistical methods, *c* is determined as a motif set or a decoy set. Otherwise, an exhaustive method is required to determine whether *c* is a motif set. In our work, to maximize the possibility that *c* is a motif set, it is determined as a motif set if *w*(*c*) ≤ *a*_*m*_; otherwise, ten substrings are removed from *c* iteratively. We use the following method to set the threshold *a*_*m*_: randomly generate 1000 samples, each containing |*c*| motif instances; then, compute the mean μ and the standard deviation σ of the weights of these samples; finally, set *a*_*m*_ to μ + σ.

12 $$ w(c)=\sum \limits_{\varphi, \varphi \hbox{'}\in c} dis\left(\varphi, {\varphi}^{\hbox{'}}\right) $$

For each obtained sample sequence set *D*’, *t*’ = |*D*’|, and the value of *q*’ is set to 0.9 to 0.95 according to the intensity of the disturbance information in the processed data. Although we maximize the possibility that *D*’ corresponds to a motif set, *q*’ cannot be set to 1. The reasons are as follows. First, the statistical method is used to determine a cluster of substrings as a motif set. Second, the distance between two substrings *φ* and *φ*’ is defined as the minimum Hamming distance between two *l*-mers *x* ∈_*l*_*φ* and *x*’ ∈_*l*_*φ*’; thus, when the distance of *φ* is calculated from different *φ*’, the *l*-mer in *φ* leading to *dis*(*φ*, *φ*’) may not come from a fixed position, which also affects the accuracy of determining a set as a motif set.

The computation time of this step is mainly determined by clustering the high-frequency substrings obtained in the previous step, i.e., the substrings stored in the set *A*’. To obtain the similarity matrix for clustering, we need to calculate the distance between each pair of substrings in *A*’ in *O*(*L*’^2^) time, where *L*’ is the average length of the substrings in *A*’. Then, given the similarity matrix, the time complexity of the AP clustering algorithm is *O*(|*A*’|^2^*r*) [30], where *r* is the number of iterations. Therefore, the time complexity of this step is *O*(|*A*’|^2^(*L*’^2^ + *r*)).

The overall time complexity of SamSelect, denoted by *T*_SamSelect_, is obtained by adding up the time complexity of the three steps of SamSelect. Since each sequence contains constant occurrences of high-frequency substrings, the number of obtained high-frequency substrings is *O*(*t*). Then, we have |*A*| = *O*(*t*) and |*A*’| = *O*(*t*). According to empirical studies, we have *L* = *O*(*l*) and *L*’ = *O*(*l*). Therefore, *T*_SamSelect_ is given as follows.

13 $$ {T}_{\mathrm{SamSelect}}=O\left(\sum \limits_{i=0}^w{4}^w\log \left|\Sigma \right|+ tn\sum \limits_{i=0}^k\left(\begin{array}{c}w\\ {}i\end{array}\right){\left(\left|\Sigma \right|-1\right)}^i+{t}^2{l}^2\right) $$

## Results and discussion

### Data, experimental setting and evaluation

Both the simulated data and real data are used in our experiment. The simulated data are generated as follows [5]: randomly generate *t n-*length DNA sequences and an *l*-length motif *m*; then, randomly select *qt* sequences, each implanted with a random instance *m*’ of *m* in a random position. The Hamming distance between *m* and *m*’ is less than or equal to *d*. To control the motif conservation, an instance *m*’ of *m* is generated as follows: randomly select *d* positions of *m*, and then, for each selected position *i*, change *m*[*i*] to a different character with probability *g*; a large *g* leads to lower motif conservation.

According to the settings of (*l*, *d*), *t*, *q* and *g*, three groups of simulated datasets are generated. The first group of simulated datasets is used to test qPMS algorithms under different (*l*, *d*) problem instances by fixing *t* = 3000 and *q* = 0.5, varying (*l*, *d*) from (9, 2) to (19, 7) and taking *g* as 0.2, 0.5 and 0.8 to represent high, intermediate and low conservation, respectively. The second group of simulated datasets is used to test qPMS algorithms under different proportions of sequences containing motif instances by fixing (*l*, *d*) = (9, 2), *t* = 3000 and *g* = 0.8 and varying *q* from 0.2 to 0.9. The third group of simulated datasets is used to test qPMS algorithms with a different scale of input by fixing (*l*, *d*) = (9, 2), *g* = 0.8 and *q* = 0.5 and varying *t* from 3000 to 10,000. For each combination of (*l*, *d*), *t*, *q* and *g*, the result is the average obtained on five randomly generated datasets.

**Fig. 3:**
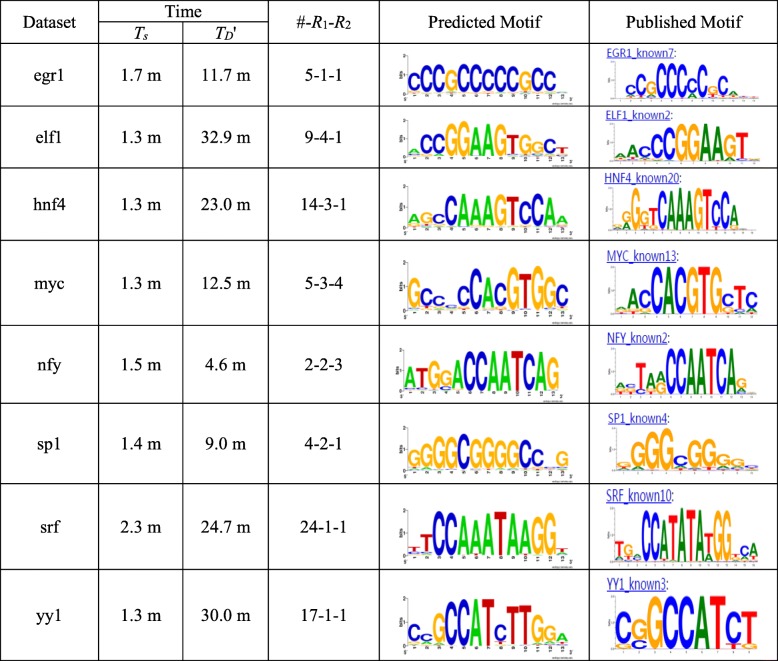
Results on the ENCODE TF ChIP-seq data. This figure shows the results on the eight *Homo sapiens* datasets selected from the ENCODE TF ChIP-seq data

**Fig. 4:**
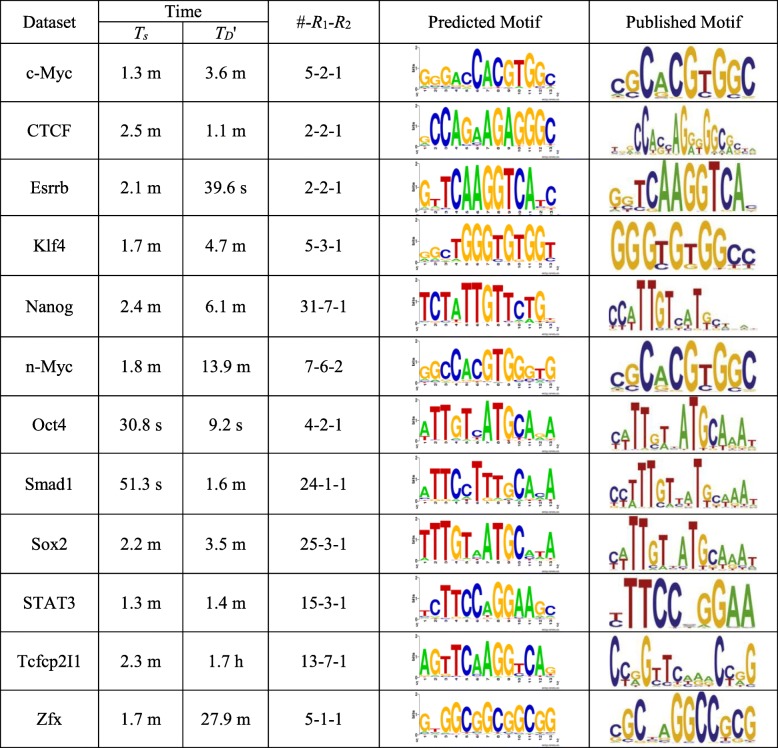
Results on the mESC data. This figure shows the results on the 12 mouse datasets in the mESC data

Eight *Homo sapiens* datasets selected from the ENCODE TF ChIP-seq data [32] and twelve mouse datasets in the mouse embryonic stem cell (mESC) data [33] are used as the real data. As shown in Tables 2 and 3, these datasets, each named for the corresponding transcription factor, have different numbers *t* of sequences, ranging from 1126 to 39,601. We use the following method to obtain the proportion *q* of sequences containing motif instances for each dataset: determine a consensus motif *m* (see the second column of Tables 2 and 3) according to the published motif (see Figs. 3 and 4), and set its value of (*l*, *d*) to a challenge problem instance [25]; then, scan the entire dataset using *m* to obtain the number *Q* of sequences containing at least one occurrence of *m* with up to *d* mismatches; finally, take *q* as *Q*/*t*. Note that, the actual value of *q* will be less than *Q*/*t* because the sequences contain random occurrences of *m*. We find that, although more sequences in ChIP-seq datasets than in traditional small datasets containing motif instances, the proportion *q* of sequences containing motif instances in ChIP-seq datasets is small. That is, a ChIP-seq dataset contains many background sequences.

For the simulated data, the stpd qPMS algorithms (FMotif [17]) and spd qPMS algorithms (TravStrR [21] and qPMS9 [25]) are tested separately to verify the effect of using the sample sequences. FMotif is designed to handle ChIP-seq datasets based on the suffix tree, whereas TravStrR and qPMS9 show good time performance when identifying motifs of large (*l*, *d*) on traditional datasets. For the real data, since the qPMS algorithms report the same results, we use a representative algorithm FMotif to verify that we can find real motifs in a reasonable time.

For each dataset *D*, the experiment uses SamSelect to select the sample sequence sets *D*’ from *D*, and then qPMS algorithms are executed separately on *D* and *D*’. When determining a sample sequence set *D*’, the number of sample sequences *t*’ is set to 100, and the proportion *q*’ of the sequences containing motif instances in *D*’ is set to 0.95 and 0.9 under the simulated and real data, respectively. Note that we use a smaller *q*’ for real data because more disturbance information is present in real data. The experimental environment is a 2.60 GHz 24-core platform with 64 Gbyte memory. SamSelect and FMotif are executed on a single core. TravStrR and qPMS9 are executed on 24 cores.

The sample sequence selection is evaluated in terms of the following two goals. The first is to compute the speedup of running time *T*_*D*_/*T*_*s*_ + *T*_*D*_’, where *T*_*s*_ is the time of selecting sample sequences using SamSelect, and *T*_*D*_ and *T*_*D*_’ are the running time of a particular qPMS algorithm on *D* and *D*’, respectively. The speedup can be fairly large as the number of sequences grows. The second is to verify whether the qPMS algorithms can find the implanted or real motifs *m* on *D*’; for FMotif, since it can output the rank of the identified motifs, we also compare the rank of *m* among the motifs obtained on *D* and that on *D*’. Note that in the case of two or more *D*’, *T*_*D*_’ is the total time on each *D*’. For the simulated data, the rank of *m* among the identified motifs is obtained on the first *D*’, since experimental results show that *m* is always present in the first *D*’; for the real data, both the rank of *D*’ containing *m* (denoted by *D*’_m_) among all *D*’ and the rank of *m* among the motifs obtained on *D*’_m_ are reported.

### Results of accelerating suffix tree-based pattern-driven qPMS algorithms

Since the maximum number of sequences processed by FMotif is limited to 3000, we only perform experiments on the first and second groups of simulated datasets, and the results are shown in Tables 4 and 5, respectively. We find that using the sample sequences selected by SamSelect to accelerate FMotif is effective. On the one hand, for each dataset *D*, the implanted motif *m* can be found on the selected sample sequence sets *D*’; in particular, the rank of *m* among the (*l*, *d*) motifs obtained on *D*’ can hold that on *D*, except for a few cases with a slight rise. On the other hand, the execution of FMotif on *D*’ achieves a good speedup (in some cases, the speedup can be more than 200); moreover, the running time of SamSelect is very small, generally negligible relative to the running time of qPMS algorithms on *D*.

**Table 4:** Results of stpd qPMS algorithms on the first group of simulated datasets

(*l*, *d*)	Conservation	*T*_*s*_	FMotif				
			*T*_*D*_	*R*_*D*_	*T*_*D*_’	*R*_*D*_’	Speedup
(9, 2)	High	33.0 s	1.6 m	1	1.2 s	1	3
	Intermediate	17.0 s	1.7 m	1	0.7 s	1	6
	Low	12.8 s	1.7 m	1	0.5 s	1	8
(11, 3)	High	26.8 s	21.1 m	1	7.0 s	1	37
	Intermediate	18.0 s	21.1 m	1	6.0 s	1	53
	Low	13.0 s	21.3 m	1	5.7 s	1	68
(13, 4)	High	28.8 s	3.0 h	1	1.0 m	1.2	119
	Intermediate	20.2 s	3.0 h	1	1.0 m	1	130
	Low	13.0 s	3.4 h	1	56.2 s	1.2	174
(15, 5)	High	29.4 s	37.7 h	1	10.4 m	1	208
	Intermediate	20.2 s	34.1 h	1	9.6 m	1	207
	Low	13.0 s	35.9 h	1	10.5 m	1	200
(17, 6)	High	29.4 s	N	N	1.7 h	1.2	> 28
	Intermediate	19.8 s	N	N	1.5 h	1	> 31
	Low	13.0 s	N	N	1.3 h	1	> 36
(19, 7)	High	32.0 s	N	N	17.3 h	1	> 3
	Intermediate	21.0 s	N	N	15.9 h	1	> 3
	Low	12.8 s	N	N	13.0 h	1	> 4

*s* seconds, *m* minutes, *h* hours, *N* no result because the running time exceeds 48 h; *T*_*s*_: running time of SamSelect; *T*_*D*_ and *T*_*D*_’: running time of a qPMS algorithm on the original dataset *D* and the sample sequence sets *D*’, respectively; *R*_*D*_ and *R*_*D*_’: the rank of the implanted motif among the identified motifs obtained on *D* and *D*’, respectively; speedup: *T*_*D*_ / *T*_*s*_ + *T*_*D*_’

**Table 5:** Results of stpd qPMS algorithms on the second group of simulated datasets

*q*	*T*_*s*_	FMotif				
		*T*_*D*_	*R*_*D*_	*T*_*D*_’	*R*_*D*_’	Speedup
0.2	13.0 s	2.4 m	1	0.5 s	1	11
0.3	13.2 s	2.2 m	1	0.5 s	1	10
0.4	13.0 s	2.0 m	1	0.5 s	1	9
0.5	13.0 s	1.9 m	1	0.6 s	1	8
0.6	13.0 s	1.2 m	1	0.5 s	1	5
0.7	14.0 s	1.1 m	1	0.5 s	1	4
0.8	14.0 s	1.0 m	1	0.7 s	1	4
0.9	14.0 s	54.9 s	1	0.5 s	1	4

*s* seconds, *m* minutes, *T*_*s*_: running time of SamSelect; *T*_*D*_ and *T*_*D*_’: running time of a qPMS algorithm on the original dataset *D* and the sample sequence sets *D*’, respectively; *R*_*D*_ and *R*_*D*_’: the rank of the implanted motif among the identified motifs obtained on *D* and *D*’, respectively; speedup: *T*_*D*_ / *T*_*s*_ + *T*_*D*_’

We perform the following further analysis according to the results. First, the use of *D*’ can effectively reduce the effect of (*l*, *d*) on the time performance of FMotif. As shown in Table 4, although the running time of FMotif increases dramatically with increasing (*l*, *d*), which is easily explained by the time complexity of the stpd qPMS algorithms, the largest (*l*, *d*) problem instances processed by FMotif within 48 h on *D* and *D*’ are (15, 5) and (19, 7), respectively. Second, the use of *D*’ can effectively reduce the effect of *q* on the time performance of FMotif. As shown in Table 5, the running time of FMotif increases as *q* decreases for *D* of the same size, whereas FMotif executed on *D*’ can have efficient and stable time performance because the sizes of *D*’ and *q*’ obtained by SamSelect are nearly fixed. Third, the speedup is relatively small when processing small (*l*, *d*) problem instances with large *q* (e.g., (*l*, *d*) = (9, 2) and *q* = 0.9). In this case, the running time of SamSelect is larger than that of the FMotif executed on *D*’ but still smaller than that of FMotif executed on *D*. Finally, as shown in Table 4, for a particular (*l*, *d*), the higher the conservation of implanted motifs the larger the running time of SamSelect. This difference occurs because a high conservation of implanted motifs leads to the accumulation of more substrings to be clustered, thus increasing the time cost of clustering.

We also perform experiments on the simulated datasets of non-challenging (*l*, *d*) instances. Except for (*l*, *d*), the settings of *t*, *q* and *g* for this group of simulated datasets are the same as those for the first group of simulated datasets. The results are shown in Table 6. We find that using the selected sample sequences to accelerate FMotif is also effective for non-challenging (*l*, *d*) instances. It should be noted that, the speedup is less than 1 for the (9, 1) instance, which is a non-challenging instance with a small (*l*, *d*). In this case, the running time of FMotif is small even on the entire dataset and it is not necessary to further accelerate FMotif using the selected sample sequences.

**Table 6:** Results on the simulated datasets of non-challenging (*l*, *d*) instances

(*l*, *d*)	Conservation	*T*_*s*_	FMotif				
			*T*_*D*_	*R*_*D*_	*T*_*D*_’	*R*_*D*_’	Speedup
(9, 1)	High	27.6 s	8.7 s	1	0.1 s	1	< 1
	Intermediate	22.8 s	7.6 s	1	0.1 s	1	< 1
	Low	15.8 s	7.7 s	1	0.2 s	1.2	< 1
(11, 2)	High	21.2 s	2.1 m	1	1.1 s	1	6
	Intermediate	15.4 s	2.0 m	1	0.9 s	1	7
	Low	12.0 s	2.1 m	1	1.0 s	1	10
(13, 3)	High	18.2 s	18.0 m	1	11.2 s	1.2	37
	Intermediate	15.0 s	18.2 m	1	10.1 s	1	43
	Low	12.0 s	18.1 m	1	9.7 s	1	50
(15, 4)	High	18.2 s	3.4 h	1	1.7 m	1.2	102
	Intermediate	15.0 s	3.4 h	1	1.6 m	1	111
	Low	11.0 s	3.3 h	1	1.5 m	1	116
(17, 5)	High	18.0 s	37.5 h	1	15.7 m	1	141
	Intermediate	15.0 s	40.6 h	1	16.3 m	1	147
	Low	10.8 s	38.8 h	1	13.8 m	1	166
(19, 6)	High	21.0 s	N	N	2.9 h	1	> 17
	Intermediate	16.2 s	N	N	2.3 h	1	> 21
	Low	10.6 s	N	N	2.1 h	1	> 22

*s* seconds, *m* minutes, *h* hours, *N* no result because the running time exceeds 48 h; *T*_*s*_: running time of SamSelect; *T*_*D*_ and *T*_*D*_’: running time of a qPMS algorithm on the original dataset *D* and the sample sequence sets *D*’, respectively; *R*_*D*_ and *R*_*D*_’: the rank of the implanted motif among the identified motifs obtained on *D* and *D*’, respectively; speedup: *T*_*D*_ / *T*_*s*_ + *T*_*D*_’

### Results of accelerating sample-pattern-driven qPMS algorithms

Tables 7, 8 and 9 give the results of testing spd qPMS algorithms (qPMS9 and TravStrR) on the first, second and third groups of simulated datasets, respectively. Since they output the same motifs as FMotif, they can also find the implanted (*l*, *d*) motifs, and thus we mainly consider their running time.

**Table 7:** Results of spd qPMS algorithms on the first group of simulated datasets

(*l*, *d*)	conservation	*T*_*s*_	qPMS9			TravStrR		
			*T*_*D*_	*T*_*D*_’	Speedup	*T*_*D*_	*T*_*D*_’	Speedup
(9, 2)	high	33.0 s	N	2.3 s	> 4895	N	0.3 s	> 5189
	intermediate	17.0 s	N	1.8 s	> 9191	N	0.2 s	> 10,047
	low	12.8 s	N	1.7 s	> 11,917	24.2 h	0.1 s	6766
(11, 3)	high	26.8 s	N	3.5 s	> 5703	N	0.6 s	> 6307
	intermediate	18.0 s	N	3.1 s	> 8190	N	0.3 s	> 9443
	low	13.0 s	N	3.0 s	> 10,800	N	0.3 s	> 12,992
(13, 4)	high	28.8 s	N	8.4 s	> 46,456	N	2.8 s	> 5468
	intermediate	20.2 s	N	7.6 s	> 6216	N	1.9 s	> 7819
	low	13.0 s	N	7.0 s	> 8640	N	1.4 s	> 12,000
(15, 5)	high	29.4 s	N	25.3 s	> 3159	N	29.5 s	> 2934
	intermediate	20.2 s	N	13.6 s	> 5112	N	10.6 s	> 5610
	low	13.0 s	N	12.5 s	> 6776	N	5.6 s	> 9290
(17, 6)	high	29.4 s	N	9.1 m	> 300	N	6.4 m	> 415
	intermediate	19.8 s	N	47.8 s	> 2556	N	36.8 s	> 3053
	low	13.0 s	N	16.1 s	> 5938	N	14.0 s	> 6400
(19, 7)	high	32.0 s	N	N	N	N	1.1 h	> 43
	intermediate	21.0 s	N	5.0 m	> 541	N	4.5 m	> 598
	low	12.8 s	N	30.7 s	> 3972	N	42.1 s	> 3148

*s* seconds, *m* minutes, *h* hours, *N* no result because the running time exceeds 48 h; *T*_*s*_: running time of SamSelect; *T*_*D*_ and *T*_*D*_’: running time of a qPMS algorithm on the original dataset *D* and the sample sequence sets *D*’, respectively; speedup: *T*_*D*_ / *T*_*s*_ + *T*_*D*_’

**Table 8:** Results of spd qPMS algorithms on the second group of simulated datasets

*q*	*T*_*s*_	qPMS9			TravStrR		
		*T*_*D*_	*T*_*D*_’	Speedup	*T*_*D*_	*T*_*D*_’	Speedup
0.2	13.0 s	N	1.3 s	> 12,084	N	0.1 s	> 13,191
0.3	13.2 s	N	1.5 s	> 11,755	N	0.1 s	> 12,992
0.4	13.0 s	N	1.7 s	> 11,755	41.8 h	0.1 s	11,490
0.5	13.0 s	N	1.7 s	> 11,755	24.3 h	0.1 s	6671
0.6	13.0 s	24.2 h	1.7 s	5919	11.2 h	0.1 s	3088
0.7	14.0 s	7.2 h	1.7 s	1651	3.1 h	0.1 s	785
0.8	14.0 s	1.5 h	1.7 s	338	1.5 h	0.1 s	377
0.9	14.0 s	9.2 m	1.7 s	35	4.1 m	0.1 s	17

*s* seconds, *m* minutes, *h* hours, *N* no result because the running time exceeds 48 h; *T*_*s*_: running time of SamSelect; *T*_*D*_ and *T*_*D*_’: running time of a qPMS algorithm on the original dataset *D* and the sample sequence sets *D*’, respectively; speedup: *T*_*D*_ / *T*_*s*_ + *T*_*D*_’

**Table 9:** Results of spd qPMS algorithms on the third group of simulated datasets

***t***	*T*_*s*_	qPMS9			TravStrR		
		*T*_*D*_	*T*_*D*_’	Speedup	*T*_*D*_	*T*_*D*_’	Speedup
3000	13.0 s	N	1.7 s	> 11,755	24.3 h	0.1 s	6671
4000	14.0 s	N	1.7 s	> 11,006	N	0.1 s	> 12,255
5000	15.0 s	N	1.7 s	> 10,347	N	0.1 s	> 11,444
6000	15.8 s	N	1.7 s	> 9874	N	0.1 s	> 10,868
7000	16.4 s	N	1.7 s	> 9547	N	0.1 s	> 10,473
8000	17.0 s	N	1.8 s	> 9191	N	0.1 s	> 10,105
9000	18.0 s	N	1.7 s	> 8772	N	0.1 s	> 9547
10,000	18.8 s	N	1.8 s	> 8388	N	0.2 s	> 9095

*s* seconds, *h* hours, *N* no result because running time exceeds 48 h; *T*_*s*_: running time of SamSelect; *T*_*D*_ and *T*_*D*_’: running time of a qPMS algorithm on the original dataset *D* and the sample sequence sets *D*’, respectively; speedup: *T*_*D*_ / *T*_*s*_ + *T*_*D*_’

On the whole, both qPMS9 and TravStrR show poor time performance on *D*, spending more than 48 h for all (*l*, *d*) problem instances except small ones with large *q*. Therefore, a large speedup on *D*’ is achieved. The use of *D*’ can effectively reduce the effects of (*l*, *d*), *q* and *t* on the time performance. Furthermore, we perform the following analysis. First, as shown in Table 7, for a particular (*l*, *d*), spd qPMS algorithms require more time to solve problem instances of high conservation because the motif instances contained in *D*’ are more similar in the case of high conservation, and too many *h* tuples are needed to generate candidate motifs. Therefore, it is not surprising that, for the case of (*l*, *d*) = (19, 7) with high conservation, qPMS9 executed on *D*’ still takes more than 48 h. Second, as shown in Table 9, the running time of SamSelect increases slightly as the data scale increases but is still very small when *t* = 10,000.

### Results on real data

We use FMotif to validate that qPMS algorithms identify real motifs by using the selected sample sequence sets *D*’. For the sake of fairness, a uniform parameter setting is used for each data set *D* in the experiments: we set *q* = 0.3, (*l*, *d*) = (13, 4) and *t*’ = 100 to execute SamSelect. After obtaining *D*’, we set *q*’ = 0.9 and use FMotif to search (13, 4) motifs in *D*’.

In Figs. 3 and 4, we give the experimental results, including the running time of SamSelect, the running time of FMotif on *D*’ and the predicted motifs. The found motif that is most similar to the published motif is taken as the predicted motif, shown in the form of a sequence logo [34]. Let *D*’_m_ denote the sample sequence set containing the predicted motif. Figures 3 and 4 also show the number of sample sequence sets obtained, the rank of *D*’_m_ (*R*_1_) and the rank of the predicted motif among the motifs present in *D*’_m_ (*R*_2_). For the real data, *R*_2_ is obtained by sorting the motifs present in *D*’_m_ in ascending order according to their enrichment *P*-value [35]. The sequence logo of the predicted motif is drawn by using the substrings similar to the motif in the entire dataset, i.e., the substrings with a Hamming distance no more than *d* / 2 from the motif. We find that FMotif executed on *D*’ can find the real motifs in a short time. It should be noted that the rank *R*_1_ and *R*_2_ differ greatly on some of the real datasets. The reasons are as follows. First, both the co-regulated motifs and the spurious motifs can disturb finding the motif to be identified. Second, the intensity of the disturbance, which affects the rank *R*_1_ and *R*_2_, is usually different for different datasets.

### Applicability of SamSelect

Our motif discovery method is not an exact algorithm. Although our method can find the implanted (*l*, *d*) motif, it does not guarantee finding all (*l*, *d*) motifs present in the entire dataset *D*. Besides the implanted (*l*, *d*) motif, some spurious (*l*, *d*) motifs may also be present in *D* by chance and are usually less conserved than the implanted motif. Our method selects sample sequences by mining high-frequency substrings, which are more likely to be the instances of highly conserved motifs. Therefore, it may miss some spurious (*l*, *d*) motifs. Moreover, some reported motifs present in the sample sequence set *D*’ may not be the (*l*, *d*) motifs present in *D*, but it is not difficult to eliminate such motifs by verifying them in *D*.

Our method is particularly designed for large DNA sequence datasets. When processing traditional datasets (*t* = 20, *n* = 600), the existing qPMS algorithms have already performed well, even for challenging (*l*, *d*) problem instances. Therefore, it is not necessary to use our method to accelerate existing qPMS algorithms on small datasets.

Moreover, the setting of *q*’ is discussed as follows. In general, the proportion *q* of sequences containing motif instances in large datasets is relatively small. For example, the maximum value of *q* for the ChIP-seq datasets given in Tables 2 and 3 is 0.68. For the sample sequence set selected by our method, the value of *q*’ is set to 0.9 to 0.95 as described in the section Methods. When *q* > 0.95, we still use our method to select the sample sequence set and set *q*’ = *q*. For a special case when *q* = 1, the reported motifs present in the sample sequence set must contain all the (*l*, *d*) motifs present in the entire dataset.

## Conclusions

To address the problem that existing qPMS algorithms are too time consuming for motif discovery on large DNA sequence datasets, we propose an algorithm to select a sample sequence set *D*’ from *D* such that *D*’ has a larger proportion of input sequences containing motif instances. Executed on *D*’, the qPMS algorithms are able to find implanted or real motifs in a significantly shorter time. In our future work, we will design the parallel version of SamSelect and the extended SamSelect algorithm for motif discovery on large alphabet datasets, e.g., protein datasets.

Notably, qPMS10 [36, 37] is also a work of sample sequence selection for the quorum planted motif search. The main difference between qPMS10 and our work is as follows. qPMS10 adopts random sampling to select a sample sequence set with *t*’ ≤ *t* and *q*’ ≤ *q*. In our work, we analyze that for a particular *t*, a small *q* will cause larger computation time. Therefore, we use word count and clustering methods to select sample sequence sets with *t*’ < *t* and 1 ≥ *q*’ > *q*.

## Abbreviations

mESC: Mouse embryonic stem cell
qPMS: Quorum planted motif search
spd: Sample-pattern-driven
stpd: Suffix tree-based pattern driven
